# The effect of weekly specialist palliative care teleconsultations in patients with advanced cancer –a randomized clinical trial

**DOI:** 10.1186/s12916-017-0866-9

**Published:** 2017-06-19

**Authors:** Patrick D. Hoek, Henk J. Schers, Ewald M. Bronkhorst, Kris C. P. Vissers, Jeroen G. J. Hasselaar

**Affiliations:** 1 Department of Anesthesiology, Pain Medicine and Palliative Care, Radboud university medical center, Nijmegen, The Netherlands; 2 Department of Primary and Community Care, Radboud university medical center, Nijmegen, The Netherlands; 3 Department of Dentistry, Radboud university medical center, Nijmegen, The Netherlands

**Keywords:** Telemedicine, Teleconsultations, Advanced cancer, Palliative care, Symptom burden

## Abstract

**Background:**

Teleconsultation seems to be a promising intervention for providing palliative care to home-dwelling patients; however, its effect on clinically relevant outcome measures remains largely unexplored. Therefore, the purpose of this study was to determine whether weekly teleconsultations from a hospital-based specialist palliative care consultation team (SPCT) improved patient-experienced symptom burden compared to “care as usual”. Secondary objectives were to determine the effects of these teleconsultations on unmet palliative care needs, continuity of care, hospital admissions, satisfaction with teleconsultations, and the burden experienced by informal caregivers.

**Methods:**

Seventy-four home-dwelling patients diagnosed with advanced cancer were recruited from outpatient clinics of a tertiary university hospital and from regional home care organizations between May 2011 and January 2015. Participants were randomized to receive weekly, prescheduled teleconsultations with an SPCT-member (intervention group), or to receive “care as usual” (control group), for a period of 12 weeks. The primary outcome of this study was: patient-experienced symptom burden indicated by the following: (1) Total Distress Score (defined as the sum of all nine subscales of the Edmonton Symptom Assessment System) and (2) the Hospital Anxiety and Depression Scale. Mixed models were used to test for differences between the two groups.

**Results:**

The Total Distress Score became significantly higher in the intervention group than in the control group, reaching significance at week 12 (adjusted difference at week 12: 6.90 points, 95% CI, 0.17 to 13.63; *P* = 0.04). The adjusted anxiety scores were higher in the intervention group than in the control group (estimate effect: 1.40; 95% CI, 0.14 to 2.55; *P* = 0.03). No difference was found between the groups in adjusted depression scores (estimate effect: 0.30; 95% CI, −1.39 to 1.99; *P* = 0.73) or in secondary outcome measures.

**Conclusions:**

Adding weekly teleconsultations to usual palliative care leads to worse reported symptom scores among home-dwelling patients with advanced cancer. Possible explanations for these findings include excess attention on symptoms and (potential) suffering, the supply-driven care model for teleconsultations used in this trial, and the already high level of specialist palliative care provided to the control group in this study.

**Trial registration:**

“The Netherlands National Trial Register”, NTR2817, prospectively registered: March 21, 2011.

## Background

Palliative care intends to improve the quality of life of patients facing life-threatening illnesses and that of their families [1]. Due to an increase in the number of patients dying from chronic, life-threatening conditions, the need for palliative care is expected to rise [2, 3]. In the Netherlands, as well as in other Western countries, the majority of these patients prefer to be cared for at home until death [4, 5]. An important condition for dying at home is the availability of easily accessible, community-based palliative home care [6–9]. Consequently, general practitioners (GPs) play a vital role in the delivery of palliative care to home-dwelling patients [8, 10].

However, when a patient’s condition deteriorates, palliative care can become increasingly complex [11]. As a result, GPs may require additional expertise [12–14]. Sustainable models for collaboration between GPs and expert palliative care teams should therefore be developed to guarantee pro-active, continuous, yet patient-centered palliative home care. Telemedicine might be an innovative approach to supporting these collaborations [15].

One of the applications of telemedicine is videoconferencing (also video- or teleconsultations), which involves the use of real-time (synchronous) audio-visual communication technology [16]. Videoconferencing has been proven to be feasible, acceptable, and effective in different fields of medicine, including psychiatry, diabetes care, and oncology [17–20]. Within palliative care, videoconferencing techniques have been used to establish multidisciplinary meetings between (rural) health centers and specialized institutes, to support patients and their families in their home-environment, and to deliver hospice care to patients living at a distance [21–26]. The results regarding the use of these techniques in palliative care are promising in terms of feasibility, acceptance and satisfaction among its users, cost-efficacy, and quality of care [22–27].

Although these results are promising, studies describing the use of videoconferencing techniques to improve the quality of palliative care for patients residing at home are generally small or have methodological limitations [16]. Recent reviews on this topic emphasize the need for randomized clinical trials (RCTs) with clinically relevant outcomes that are measured with validated instruments [16, 28–30].

Therefore, the primary objective of this study was to evaluate whether weekly teleconsultations between patients receiving palliative home care and a hospital-based specialist palliative care consultation team (SPCT) improves patient-experienced symptom burden when compared to “care as usual”.

The secondary objectives were to evaluate the effect of these teleconsultations on (1) unmet palliative care needs, (2) experienced continuity of medical care, (3) hospital admissions, and (4) satisfaction with teleconsultations. Furthermore, the effect of teleconsultations on the burden experienced by informal caregivers, as well as healthcare professionals’ satisfaction with teleconsultations, were evaluated.

## Methods

### Design

We conducted a two-armed, non-blinded randomized clinical trial. The study protocol (Reg 2010/382) was approved by the Committee on Research Involving Human Subjects Region Arnhem-Nijmegen and has been previously published [15]. The study was prospectively registered at The Netherlands National Trial Register (NTR2817). During the study period, two amendments were approved by the Committee on Research Involving Human Subjects. Written informed consent was obtained from all participants.

### Participants

#### Setting and location

Initially, the inclusion period for this trial was 18 months. However, mainly as a result of recruitment delay, the inclusion period was extended to 45 months (May 2011–January 2015). Patients were recruited from the outpatient clinics of the Radboud university medical center, Nijmegen, the Netherlands, mainly at the Department of Palliative Medicine as well as from regional home care organizations.

#### Inclusion criteria

Participants had to be aged 18 years or above, Dutch speaking, and able to give informed consent. Furthermore, they initially had to meet the following criteria: (1) be diagnosed with a progressive oncological condition, (2) reside at home, (3) have a GP who agrees to participate, (4) have a Karnofsky Performance Status score (KPS) of 60 or below, and (5) have a life-expectancy of 3 months or less.

Because of recruitment problems, the first amendment was implemented in February 2013, after 24 participants had been included. The criteria of a KPS of 60 or below and life expectancy of 3 months or less were replaced by a new criterion, namely that patients should not be receiving any disease-modifying treatment at the time of inclusion nor would do so in the future. However, as recruitment did not improve sufficiently, a second amendment was implemented in July 2013, after 35 participants had been included, whereby the latter criterion was removed and was not replaced with new inclusion criteria.

### Randomization

After completing the baseline measurements, the participants were randomized into two groups: intervention or control. We used block randomization with different size blocks (4 and 6) to maintain an equal balance between groups. Randomization occurred at the level of individual patients, with an allocation ratio of 1:1. The author involved in the process of approaching, informing and visiting participants (PH) was not informed about the outcome of the randomization process before baseline measurements had taken place.

Initially, it was expected that patients would be recruited by their GPs and therefore, to prevent bias, a cluster randomization procedure at the level of the GP was described in the original study protocol [15]. However, the vast majority of patients were eventually recruited via the SPCT and GPs did not recruit individual participants for this study. Consequently, there were no clusters of participants with the same GP. Therefore, in the first amendment, we decided that there was no further need for cluster randomization. As a result, randomization took place at the level of the individual participant.

### Care as usual

Participants in both groups received palliative home care provided by their GP, supported by the SPCT according to the standard referral procedures, i.e., patients could be referred to the SPCT by their GP or by the attending hospital specialist or were not referred at all. If applicable, follow-up by the SPCT occurred by phone or by patients visiting the outpatient clinic, depending on the patient’s preference, the complexity of their problems, and/or the stage of their disease.

### Intervention group

#### Procedure

Participants in the intervention group had weekly teleconsultations for a period of 13 weeks in addition to their usual care. First, a teleconsultation device was installed at the patient’s home. Patients who had not visited the SPCT before were evaluated at the outpatient clinic or during a home visit by one of the SPCT members (a nurse or physician). Then, teleconsultations were scheduled on a weekly basis for a period of 13 weeks. At the agreed time, a member of the SPCT (mostly the nurse practitioner) initiated the teleconsultation. In between these scheduled appointments, the participants could not directly contact the SPCT members through teleconsultation. When in need of medical advice, the patients were encouraged to contact their GP; however, if necessary, the SPCT could be reached by phone. A predefined consultation schedule was available to the SPCT members to ensure that all domains of palliative care were sufficiently covered during the teleconsultations. Problems and needs of participants were identified and discussed with other team members if necessary. The participant’s GP was invited to visit and join the patient during the teleconsultation appointments. If this was not possible, after the first teleconsultation, a member of the SPCT contacted the patient’s GP by phone to discuss the patient’s current problems and needs, possible treatment policies, and the GP’s involvement during the following study period. After the first and the last scheduled teleconsultation, the SPCT was encouraged to send a letter to the participant’s GP, outlining the patient’s current problems and needs and advised treatment policies.

#### Teleconsultation device

Initially, in 2011, teleconsultation devices consisted of a Pal4-desktop computer (“Bidibox”, Focuscura Inc., the Netherlands) with a touch screen, a separate microphone/speaker, and a separate camera. During the study period, tablet computers became available. These devices seemed more user-friendly, and therefore the “Bidibox” computers were replaced by tablet computers (iPad 2® and iPad mini®; Apple Inc., United States). The Pal4 application was replaced by FaceTime® (Apple Inc., United States).

### Outcome measures

The primary outcome was patient-experienced symptom burden, based on the Edmonton Symptom Assessment System (ESAS) and the Hospital Anxiety and Depression Scale (HADS). The secondary and other study outcomes have been described in our study protocol [15].

### Data collection

Data collection ended after 12 weeks. During the 13th week of the study period a closing teleconsultation was scheduled for participants from the intervention group. During this 13th week participants did not complete any questionnaires.

Questionnaires for baseline measurements were handed over by one of the researchers (PH) or sent through postal mail. After completion, participants handed over the questionnaires to the researchers or sent them back through postal mail. During the study period, participants received and returned the required questionnaires through postal mail and sent them back every 4 weeks. If necessary, one of the researchers (PH) reminded the participants by phone, SMS, or e-mail to return the required questionnaires.

Participants completed the following questionnaires: the ESAS (at baseline, weekly follow-up), HADS, Problems and Needs in Palliative Care-short version, and a modified version of the Nijmegen Continuity Questionnaire (NCQ) (all three: at baseline, four-weekly follow-up). Informal caregivers completed one questionnaire: Self-Perceived Burden from Informal Care (EDIZ) (at baseline, two-weekly follow-up).

Additionally, participants in the intervention group completed a Patient Satisfaction Questionnaire (PSQ) after the first two teleconsultations. If applicable, the SPCT members involved in the teleconsultations and the participant’s GPs also completed a PSQ after the first two teleconsultations.

Finally, demographic information was collected at baseline. Information on other study outcomes (GP contacts, complex interventions, and hospital admissions [15]) was requested from the patient’s GP after the study period.

#### Questionnaires

The ESAS is a self-reporting scale consisting of nine symptoms that are common in patients diagnosed with cancer [31]. Items can be scored on a 0–10 visual numerical scale (with 0 indicating the absence of a symptom and 10 indicating the worst imaginable intensity of a symptom). The ESAS is widely used, and its psychometric properties are considered good in our study population [32–35]. The Total Distress Score (TDS) is defined as the sum of the nine subscales. The HADS is a 14-item self-report screening scale that provides an indication of the possible presence of anxiety and depressive symptoms [36]. Each item is scored on a 4-point Likert scale. The questions assess symptoms in the preceding week. Its psychometric properties are considered moderate to good [35, 37].

The secondary outcomes of this study were measured by the following questionnaires: (1) the Problems and Needs in Palliative Care-short version (patient-experienced problems and needs) [38, 39], (2) the NCQ (patient-experienced continuity of care) [40, 41], (3) the PSQ (satisfaction with teleconsultations) [42–44], and (4) the Self-Perceived Burden from Informal Care [45].

#### Sample size

For the original calculation of sample size, we refer to our study protocol [15]. As a result of the aforementioned changes in the randomization procedure, we removed the correction factor to adjust for a cluster effect, yielding an aimed total sample size of 84 patients.

### Statistical analysis

Data were stored and analyzed in the Radboud university medical center, Nijmegen, the Netherlands, using SPSS Software (IBM Corp. Released 2011. IBM SPSS Statistics for Windows, Version 20.0. Armonk, NY: IBM Corp).

Observed values were reported as the mean and standard deviation (SD) for continuous variables, and the number and percentage for categorical variables.

The null hypothesis for this study was that there would be no difference in patient-experienced symptom burden between the intervention and control groups. To test this hypothesis, for all outcomes, a mixed model with a random intercept for “Patient” was used to accommodate the repeated measurements over time. The dependent variable was the relevant outcome, at any time after T0. For scale variables, a linear mixed model was used, while for dichotomous outcomes, a generalized mixed model with a logit-link function was used. To identify the best model, a series of models was tested. Starting from the simplest model, each subsequent model was extended step by step until further extensions showed non-significant improvement.

The simplest model had only the Experimental condition (i.e., “group”: intervention or control) and the measurement of the outcome variable at T0 (“score at baseline”) as the independent variables. The next step added Time as an independent variable. After that, the interaction between Experimental condition and Time (“group*time”) was considered.

By using mixed models, every available observation contributes to the modeling of the relation between outcome and variables. As a result of this approach, missing data did not result in exclusion of participants from analyses.

Statistical software R, version 3.0.1, was used in combination with the *lmer* procedure from the *lme4* library for the mixed modeling analyses.

## Results

**Fig. 1:**
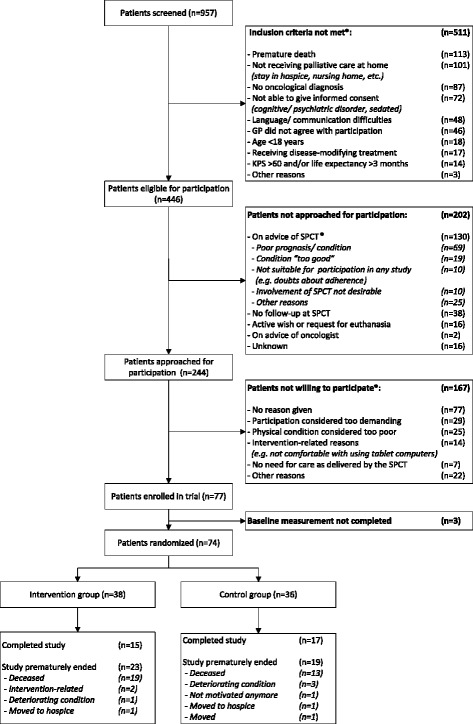
Screening and participants, *More than one reason may apply

A total of 957 patients were screened. We excluded patients who (1) did not meet the inclusion criteria (n = 511); (2) were not approached for participation for other reasons (n = 202); or (3) were not willing to participate (n = 167). Thus, a total of 77 participants gave informed consent and were enrolled in the study. Of these participants, three did not complete the baseline measurement. Consequently, 74 participants were randomized to either the intervention group (n = 38) or the control group (n = 36). A total of 32 participants (42%) completed the study. All other participants prematurely ended this study for several reasons, mostly related to death or deteriorating condition (Fig. 1). The attrition rates were relatively high in both groups; 61% and 53% of participants in the intervention and control group, respectively, stopped contributing data during the course of the study. These rates did not differ between groups (*P* = 0.64). Sensitivity analyses on the attrition rates in relation to baseline measurements of participants showed a significant correlation between baseline scores on TDS, HADS-anxiety, and HADS-depression and attrition rates for participants from the control group. The effect of this on the study outcomes was mitigated by including baseline scores as independent variables in the regression models.

Demographic baseline characteristics are shown in Table 1. All 74 participants were analyzed for the primary outcome. Furthermore, 71 participants had an informal caregiver who gave informed consent and was enrolled in the study.

**Table 1:** Baseline characteristics of the participants (n = 74)

	Intervention group (n = 38)		Control group (n = 36)	
Demographics				
Age, mean (SD), years	62.3	(9.0)	61.9	(10.6)
Female, n (%)	11	(29)	14	(39)
Diagnosis, n (%)				
Urogenital cancer	13	(34)	15	(42)
Gastro-intestinal cancer	6	(16)	5	(14)
Hepatobiliary and pancreatic cancer	4	(11)	5	(14)
Lung cancer	6	(16)	2	(6)
Head and neck cancer	3	(8)	5	(14)
Breast cancer	3	(8)	0	(0)
Skin cancer	0	(0)	1	(3)
Other type of cancer	3	(8)	3	(8)
Marital status, n (%)				
Married/permanent relationship	27	(71)	29	(81)
Divorced	5	(13)	1	(3)
Single	4	(11)	5	(14)
Widow(er)	2	(5)	1	(3)
Having one or more children, n (%)	32	(84)	30	(83)
Living situation, n (%)				
Together with partner and/or children	28	(74)	29	(81)
Alone	9	(24)	6	(17)
Other living situation	1	(3)	1	(3)
Household, mean number of persons (SD)	2.0	(0.9)	2.0	(0.6)
Highest educational level, n (%)				
No education/primary school	1	(3)	3	(8)
Lower vocational education	10	(26)	7	(19)
Lower general secondary education	4	(11)	8	(22)
Intermediate vocational education	11	(29)	7	(19)
Higher general secondary education/pre-university education	3	(8)	2	(6)
Higher professional education/university	9	(24)	9	(25)

Percentages may not add to 100% due to rounding

*SD* Standard deviation

Due to financial and time constraints this study was ended after 45 months before the calculated sample size was reached.

### Symptom burden

**Fig. 2:**
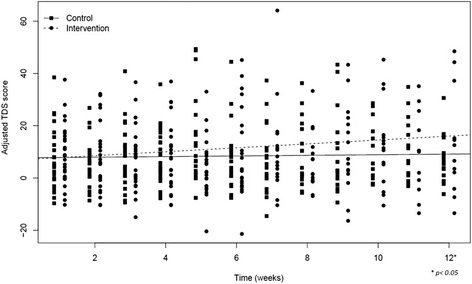
Adjusted Total Distress Score during the study period

At baseline, the mean observed TDS in the intervention group was almost 7 points higher (31.03 ± 17.21) than that of the control group (24.33 ± 14.54). Over the study period, the control group showed a slight decline in the mean observed TDS. The intervention group also showed a decline in the mean observed TDS during the first 8 weeks of the study; however, in the last 4 weeks, the mean TDS increased almost 9 points to 36.62 (±20.14) at week 12, compared to 22.38 (±11.27) in the control group (Table 2). The adjusted TDS scores became significantly higher in the intervention group, indicating a growing symptom burden in this group over time compared to the control group (Fig. 2). When testing specific points in time, this difference reached significance at week 12 (adjusted difference at week 12: 6.90; 95% CI, 0.17 to 13.63; *P* = 0.04; Table 3).

**Table 2:** Observed values at baseline and weeks 4, 8, and 12

		Group	Baseline	Week 4	Week 8	Week 12
ESAS						
TDS, mean (SD)	(0–90)	Intervention	31.03 (17.21) n = 38	30.68 (19.58) n = 22	27.73 (15.87) n = 15	36.62 (20.14) n = 13
		Control	24.33 (14.54) n = 36	24.17 (13.79) n = 27	22.20 (10.89) n = 20	22.38 (11.27) n = 16
HADS						
Anxiety, mean (SD)	(0–21)	Intervention	7.24 (4.70) n = 38	7.48 (4.19) n = 23	7.11 (3.39) n = 16	8.46 (4.25) n = 13
		Control	6.22 (3.91) n = 36	5.23 (3.41) n = 26	4.71 (3.08) n = 21	5.06 (3.21) n = 16
Depression, mean (SD)	(0–21)	Intervention	7.66 (3.87) n = 38	7.45 (4.82) n = 23	7.31 (4.45) n = 16	7.85 (5.10) n = 13
		Control	6.49 (4.57) n = 36	6.25 (4.16) n = 26	5.76 (3.92) n = 21	7.00 (4.95) n = 16
PNPC-sv						
Number of unmet needs, mean (SD)	(0–32)	Intervention	3.94 (5.68) n = 37	2.07 (3.82) n = 23	1.31 (3.48) n = 16	2.02 (3.88) n = 13
		Control	2.92 (4.36) n = 36	2.57 (3.75) n = 25	1.42 (2.60) n = 17	2.80 (5.21) n = 15
NCQ						
Personal continuity, mean (SD)	(6–30)	Intervention	24.33 (3.76) n = 36	24.52 (3.10) n = 23	24.73 (3.26) n = 15	24.38 (3.55) n = 13
		Control	22.81 (4.43) n = 33	23.00 (3.43) n = 25	23.28 (5.33) n = 18	21.92 (4.27) n = 13
Team continuity (within hospital), mean (SD)	(4–20)	Intervention	14.20 (3.26) n = 30	15.89 (2.27) n = 18	15.75 (2.01) n = 12	14.60 (3.24) n = 10
		Control	15.12 (3.15) n = 31	15.59 (2.92) n = 17	15.42 (2.61) n = 12	13.73 (2.45) n = 11
Cross-boundary continuity, mean (SD)	(4–20)	Intervention	15.83 (3.09) n = 24	16.56 (3.01) n = 18	14.20 (2.78) n = 15	16.59 (2.98) n = 13
		Control	15.16 (3.13) n = 25	15.35 (2.57) n = 18	14.10 (4.25) n = 13	14.33 (3.20) n = 9
EDIZ						
Total score, mean (SD)	(9–45)	Intervention	15.78 (5.87) n = 36	14.54 (7.09) n = 21	16.09 (8.39) n = 16	15.95 (8.25) n = 12
		Control	15.54 (6.46) n = 35	17.45 (8.39) n = 25	14.74 (5.88) n = 19	15.00 (7.10) n = 14

*SD* standard deviation, *ESAS* Edmonton Symptom Assessment System, *TDS* Total Distress Score, *HADS* Hospital Anxiety and Depression Scale, *PNPC-sv* Problems and Needs in Palliative Care-Short Version, *NCQ* Nijmegen Continuity Questionnaire, *EDIZ* self-perceived burden from informal care

**Table 3:** Primary outcome measures – mixed models

	B (SE)	95% CI		*P* value
		Lower limit	Upper limit	
ESAS-TDS				
Intercept	7.26 (3.17)			
Group^a^	−0.66 (3.26)	−6.99	5.66	0.84
TDS score at baseline	0.78 (0.11)	0.57	0.99	<0.001
Time^b^	0.12 (0.17)	−0.21	0.45	0.48
Group*time	0.63 (0.25)	0.14	1.11	0.01
HADS-A				
Intercept	0.54 (0.76)			
Group^a^	1.40 (0.65)	0.14	2.66	0.03
HADS-A score at baseline	0.78 (0.09)	0.61	0.95	<0.001
Time^b^	0.12 (0.06)	0.01	0.23	0.04
HADS-D				
Intercept	1.56 (1.03)			
Group^a^	0.30 (0.87)	−1.39	1.99	0.73
HADS-D score at baseline	0.70 (0.11)	0.49	0.91	<0.001
Time^b^	0.13 (0.08)	−0.03	0.29	0.12

*B* estimate effect, *SE* standard error, *95% CI* 95% confidence interval, *ESAS* Edmonton Symptom Assessment System, *TDS* Total Distress Score, *HADS-A* Hospital Anxiety and Depression Scale – Anxiety, *HADS-D* Hospital Anxiety and Depression Scale – Depression

^a^Group: intervention = 1; control = 0

^b^Time: week number, baseline = week 0

The mean observed HADS-anxiety scores in the control group declined somewhat during the first 4 weeks of the study (from 6.22 ± 3.91 to 5.23 ± 3.41) and thereafter remained rather stable. Within the intervention group, the mean observed anxiety scores were relatively stable during the first 8 weeks of the study, but increased between week 8 and week 12 from 7.11 (±3.39) to 8.46 (±4.25). Within the intervention group, the mean observed HADS-depression scores remained stable during the study period. The mean observed depression scores declined in the control group during the first 8 weeks (from 6.49 ± 4.57 to 5.76 ± 3.92); however, over the last 4 weeks, depression scores increased to 7.00 (±4.95) at week 12 (Table 2). The adjusted anxiety scores were significantly higher in the intervention group than in the control group (estimate effect: 1.40; 95% CI, 0.14 to 2.66; *P* = 0.03). The depression scores did not differ between the two groups (estimate effect: 0.30; 95% CI, −1.39 to 1.99; *P* = 0.73; Table 3).

### Secondary and other study outcomes

The mean number of unmet needs did not differ between the intervention and control groups (estimate effect: –0.01; 95% CI, −0.07 to 0.04; *P* = 0.67). Additionally, the number of participants having at least one unmet need did not differ between the groups (OR: 0.79; 95% CI, 0.19 to 2.92; *P* = 0.66). On all three subscales of the NCQ for continuity of care, i.e., personal continuity (estimate effect: 0.15; 95% CI, −0.09 to 0.38; *P* = 0.22), team continuity (estimate effect: 0.16; 95% CI, −0.20 to 0.51; *P* = 0.39), and cross-boundary continuity (estimate effect: 0.29; 95% CI, −0.08 to 0.67; *P* = 0.13), there were no differences between groups. Finally, the mean number of hospital admissions during the study period did not differ between the intervention group (0.47) and the control group (0.38; *P* = 0.60). Study outcome measures regarding GP contacts and complex interventions did not statistically differ between both groups. The mean satisfaction scores after the first two teleconsultations were high for both the participants (90.4 ± 8.2 and 89.4 ± 9.7) and the SPCT members (87.0 ± 7.1 and 85.5 ± 15.2).

When comparing the number of informal caregivers with a high perceived burden (defined as the upper third of the group), there was a trend towards a lower proportion of informal caregivers with a high perceived burden in the intervention group (estimate effect: −2.24; 95% CI, −5.24 to 0.02; *P* = 0.05).

## Discussion

### Main results

We found a difference in reported symptom burden between home-dwelling patients with advanced cancer receiving palliative care “as usual” and patients who additionally had weekly teleconsultations with a hospital-based SPCT. Therefore, we can reject our null hypothesis, which stated that there would be no difference in patient-experienced symptom burden between the intervention group and the control group. Contrary to our expectations, this additional intervention led to a higher reported symptom burden in the intervention group than in the control group. The number of unmet needs, experienced continuity of care, and reported hospital admissions did not differ between groups.

#### Comparison with the literature

To the best of our knowledge, this is the first RCT to show that, despite difficulties in recruitment, technical challenges, and relatively high drop-out rates, it is possible to perform an RCT on telemedicine, which can be considered a complex intervention, in palliative care [46].

We found an observed mean TDS ranging from 21.4 to 36.6. This is comparable to other studies reporting ESAS scores from patients with advanced cancer visiting outpatient clinics [47–49], although higher scores have been reported [50].

While the TDS in the control group remained relatively stable during the study period, the TDS of the intervention group increased. For both the observed and the adjusted TDS, this increase was of more than 4 points, indicating a clinically relevant deterioration according to Hui et al. [49]. This deterioration seems to be in contrast with earlier research in which video technologies did not affect symptom burden or even led to a possible improvement in clinical outcomes [21, 25]. On the other hand, the adjusted difference between both groups at week 12 was less than 8 points, which is the minimum clinically important difference stated in the power calculation of our study protocol [15]. Therefore, and given the relatively wide confidence intervals of the adjusted difference in TDS, the clinical relevance of the difference in TDS should be interpreted with proper caution.

The higher reported symptom burden found in this study might be partially explained by the participant’s perceptions of symptoms. Participants in the intervention group received weekly attention for their (potential) symptoms. This might have led to a higher awareness of symptoms among participants, leading to a worsening symptom experience. This phenomenon is referred to as the “nocebo effect” [51]. In a review on this topic, Häuser et al. [51] state that patients are “*highly receptive to negative suggestion, particularly in situations perceived as existentially threatening*”. This negative suggestion includes focusing one’s attention towards the presence of particular symptoms. The “nocebo effect” may be avoided by adapting the frequency and timing of the teleconsultations to the actual needs of the individual patient.

Another explanation for our findings might be the differential recall bias among participants. For participants in the intervention group, the reported symptom burden was strongly related to the content of their weekly teleconsultations. As a result, their symptom burden may have been registered more precisely, possibly leading to higher symptom scores.

### Strengths and limitations

An important strength of this study is that we have systematically screened a large group of patients for participation in this trial. Despite considerable difficulties in recruitment, the intended sample size was nearly reached, although over a longer period of time and with adjusted inclusion criteria. Finally, this is the first completed RCT on telemedicine in palliative care with outcome measures that are clinically relevant and relate directly to patient care and experienced quality of life. This study also has some limitations. First, a considerable group of patients who were eligible for participation in this trial were not approached, mostly as a result of clinical considerations, which may have caused non-differential selection bias. Additionally, the relatively small number of patients who were eventually approached for participation might also reflect that offering teleconsultations in the context of a randomized study might not fit the needs of palliative care patients. Second, the attrition rate in this study was relatively high and attrition may depend on the clinical condition of participants. Although in our statistical models we have corrected for baseline measurements (i.e., the clinical condition of participants at the start of the study), study outcomes may have been influenced by participants’ worsening clinical condition during the study. Third, the participants sometimes had difficulty adequately completing the questionnaires as a result of their varying clinical conditions. This might have led to information bias, although likely non-differential. Fourth, the outcome measure “place of death” was described in the study protocol, however, it was not included in the information request at the patient’s GP; thus, this study outcome is missing. Fifth, two amendments had to be made to the study protocol to improve the recruitment rates. As a result of widening the inclusion criteria of this study, the study population may have become more heterogeneous, which may have led to a dilution of the effect of the intervention. Finally, the involvement of GPs in this study was less than expected; therefore, the participants were not recruited by their GP but were instead recruited at the outpatient clinic of the SPCT, probably leading to higher levels of specialist care in both groups, which may have positively affected symptom scores.

### Clinical implications

In this study, we introduced a model for teleconsultation in palliative care that was intended to be proactive and was mainly supply driven. Teleconsultations were scheduled on a weekly basis for a period of 3 months, irrespective of the actual needs of the patient regarding the timing and frequency of these teleconsultations. This model was shown to be ineffective in reducing experienced symptom burden, even though patients and caregivers showed a high degree of satisfaction. Therefore, we propose focusing on care models that are patient-tailored and demand-driven, i.e., patients themselves indicate when they are in need of palliative care (tele)consultations. This model could prevent a possible excess of medical care regarding palliative care, death, and dying. At the same time, to avoid a rather reactive palliative care approach, patients should be provided tools and support to guide them in proactively contacting their caregivers when problems arise.

## Conclusions

Telemedicine is emerging in all fields of medicine, including palliative care. Despite promising earlier research, the present study shows that telemedicine does not necessarily lead to a better quality of advanced cancer care. Indeed, the use of telemedicine might create a situation in which patients experience a higher symptom burden, despite high degrees of satisfaction. Future research and care models should therefore explore the beneficial as well as the potentially harmful aspects of teleconsultations within advanced cancer care, thereby focusing on (1) ways to optimize multidisciplinary care via teleconsultations, (2) the appropriate timing and frequency of palliative care teleconsultations for patients with advanced cancer and other groups of vulnerable patients, (3) possibilities for patient-tailored, demand-driven teleconsultations, and (4) the potential impact of technology as such on the patient’s sense of wellbeing. These issues should be adequately addressed, both in future research and in implementation trajectories regarding the use of telemedicine in palliative home care.

## Abbreviations

95% CI: 95% confidence interval
ESAS: Edmonton Symptom Assessment System
GP: general practitioner
HADS: Hospital Anxiety and Depression Scale
KPS: Karnofsky Performance Status score
NCQ: Nijmegen Continuity Questionnaire
PSQ: Patient Satisfaction Questionnaire
RCT: randomized clinical trial
SPCT: specialist palliative care consultation team
TDS: Total Distress Score
